# Spatiotemporal tracing of pandemic spread from infection data

**DOI:** 10.1038/s41598-021-97207-5

**Published:** 2021-09-03

**Authors:** Satyaki Roy, Preetom Biswas, Preetam Ghosh

**Affiliations:** 1 Department of Genetics, University of North Carolina, Chapel Hill, USA; 2 Arizona State University, Tempe, AZ USA; 3 Department of Computer Science, Virginia Commonwealth University, Virginia, USA

**Keywords:** Computational models, Mathematics and computing, Computer science

## Abstract

COVID-19, a global pandemic caused by the Severe Acute Respiratory Syndrome Coronavirus 2 virus, has claimed millions of lives worldwide. Amid soaring contagion due to newer strains of the virus, it is imperative to design dynamic, spatiotemporal models to contain the spread of infection during future outbreaks of the same or variants of the virus. The reliance on existing prediction and contact tracing approaches on prior knowledge of inter- or intra-zone mobility renders them impracticable. We present a spatiotemporal approach that employs a network inference approach with sliding time windows solely on the date and number of daily infection numbers of zones within a geographical region to generate temporal networks capturing the influence of each zone on another. It helps analyze the spatial interaction among the hotspot or spreader zones and highly affected zones based on the flow of network contagion traffic. We apply the proposed approach to the daily infection counts of New York State as well as the states of USA to show that it effectively measures the phase shifts in the pandemic timeline. It identifies the spreaders and affected zones at different time points and helps infer the trajectory of the pandemic spread across the country. A small set of zones periodically exhibit a very high outflow of contagion traffic over time, suggesting that they act as the key spreaders of infection. Moreover, the strong influence between the majority of non-neighbor regions suggests that the overall spread of infection is a result of the unavoidable long-distance trips by a large number of people as opposed to the shorter trips at a county level, thereby informing future mitigation measures and public policies.

## Introduction

COVID-19, caused by the Severe Acute Respiratory Syndrome Coronavirus 2 and declared a global pandemic by the World Health Organization, has claimed millions of lives and disrupted social and economic order^1^. With a death toll of over 4 million worldwide, most countries are straddling the existential question on whether lockdown or the resulting poverty will claim more lives^2^. The infectious disease scientists believe that large-scale vaccination campaigns may help achieve herd immunity and restore normalcy by the end of 2021^3^. While vaccines are expected to check contagion and save over 3 million people from losing their livelihood, there are concerns about the limited manufacture and distribution as well as the debilitating physiological effects of the newer variants of the virus^4^.

There is consensus in the infectious disease research community that COVID—the third coronavirus outbreak in the last two decades^5^—is here to stay, impelling widespread behavioral changes with regard to social mixing and prompt government policymaking^6^. It is imperative to not only prepare for a rapidly changing socioeconomic and demographic landscape in the post-COVID-19 world, but also build an epidemiological knowledge base that can inform decision-making on the basis of the current trends like the susceptibility, duration of immunity of immunized population to the virus, effect of seasonality on spread, etc. Models used to build such knowledge bases must be dynamic, adaptive and incorporate the spatial and temporal context to be able to make more accurate predictions on spread.

Existing studies discuss factors, symptoms and preventive measures of COVID-19^7^. Machine learning-based prediction models leverage epidemiological and clinical data to identify vulnerable individuals^8,9^, trace the trends in infection dynamics^10^ and measure the long-term effects of testing in identifying affected individuals^11^. We proposed a time-varying linear optimization-based approach, which incorporated epidemiological factors, like population density, susceptible count and infected ratio as well as transportation costs, to distribute vaccines among zones^12^ and optimization measures based on network science to guide human mobility and restrict contact of susceptible and infective individuals^13^. Regression and topic models have been used to pinpoint socioeconomic factors controlling contagion and the economic sectors affected by it^14,15^, while reinforcement learning has been employed to design a dynamic pandemic lockdown strategy to control mobility of individuals within zones based on its healthcare resource budget^16^.

There have been efforts to trace the path of the pandemic to predict and mitigate contagion^17^. All these models rely heavily on the knowledge of inter- and intra-zone mobility patterns. For instance, first, Ahmed et al. study the functioning of contact tracing apps based on proximity and duration of contact with infected individuals^18^, while *InfluenzaNet* and *Flutracking* build a repository from online surveys on geographic location of patients^19,20^. By examining the parameters of the Susceptible–Exposed–Infected–Recovered (SEIR)-like transmission model on a network of 107 provinces characterized by high inter-zone mobility, Gatto et al. analyzed the effects of intervention on the diffusion of infection from hotspot zones^21^. However, it is still infeasible to possess a prior (satellite- or mobile application survey-based) knowledge of mobility under all circumstances.

Network and population-based models show that human mobility, especially long-distance travels, has been deemed as a significant cause for contagion. Ohsawa et al. have characterized the effect of travel on infection spread through Susceptible-Exposed-Infected-Recovered-Susceptible (SEIRS) model on newly infected cases, where they considered susceptible individuals contracting the disease from locally infected people or travelers^22^. Verma et al. employed daily infection and mortality numbers of six nations to demonstrate the effects of mobility restriction on contagion^23^. Livatiodis also utilized the daily infected numbers in the US and Italy to elucidate the relationship between environmental temperature and infection spread^24^. Barreiro et al. highlighted the importance of time lapse between identification and isolation of infected individuals in modeling spread dynamics^25^.

**Contributions.** We present a spatiotemporal approach to trace the path of the COVID-19 pandemic by leveraging a network inference algorithm (called *GENIE3*) solely based on the daily infection counts of the different zones comprising a region. As evidenced by the existing literature, the daily infection count over time is considered to be a comprehensive measure of the extent of infection spread. This is because it is difficult to quantify the real asymptomatic (or exposed) count, while the mortality rates are contingent on several socioeconomic and demographic covariates^14,26^. The proposed approach generates a complete network of zones (viz., counties, boroughs, states, etc.), where the weight of each directed link measures the strength of influence of one zone on another. It factors in *time* by tracing the evolution of the influence of zones through a sliding window considering a prespecified time interval of infection count, while it addresses the *spatial* aspect by quantifying the interaction (or absence thereof) among neighboring zones that affect contagion. Our analysis identifies zones, called *spreader zones*, that posses strong outgoing links in the influence networks over time. While we show these zones to contribute significantly to contagion, they may or may not be drivers of infection (or *causal*) in nature.

We demonstrate the efficacy of the approach on a micro scale of the counties in New York as well as the macro scale of the states of USA, while studying the variations in spread dynamics in regions varying in size. We employ *cosine similarity* and a variant of *topological sorting* to derive directed acyclic graphs to measure shifts in interaction pattern among zones as well as the trajectory of contagion during the early phases of the pandemic. Moreover, we identify specific zones that (1) act as major hotspots (or *spreaders*) and also the *most affected zones* at different timepoints, based on their inflow and outflow of network contagion traffic; and (2) exhibit the highest and least variation in interaction with other zones. Finally, we collate our findings to trace the likely path of the pandemic, analyze the role human mobility has had on the early and later waves, and infer ways to contain future spread.

## Materials and methods

### Graph theory preliminaries

A graph is an ordered pair $G = (V, E)$ where *V* is a finite, non-empty set of objects called *vertices* (or nodes); and *E* is a (possibly empty) set of 2-subsets of *V*, called *edges*^27^. A directed graph is a graph in which edges have directions. A directed edge $(u,v) \in E$, allowing unidirectional information flow from vertex *u* to *v* and not necessarily from *v* to *u*. Each node $u \in V$ has *in-neighbors* defined as a set of nodes *v* such that there exists an edge from *v* into *u*, i.e., $e(v, u) \in E$. Similarly, the *out-neighborhood* of $u \in V$ consists of nodes *v* such that there exists an edge *u* into *v*, i.e., $e(u, v) \in E$. In a weighted directed graph, $(u, v) \in E$ is associated with a weight $w_{u,v} \in [0, 1]$, which is measure of the strength of influence of *u* on *v*.

**Figure 1:**
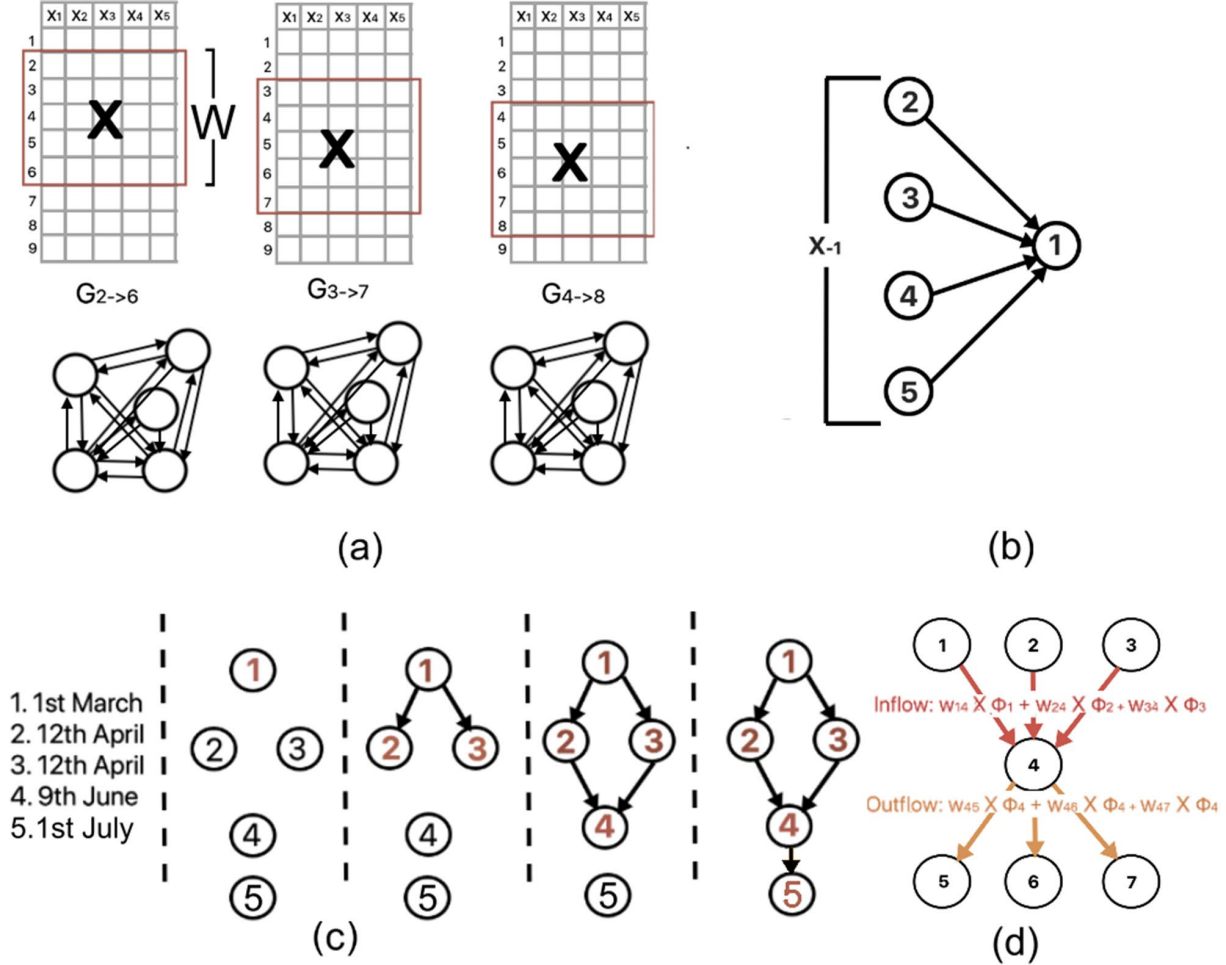
Graph structures. (**a**) Temporal influence networks created from matrix $\mathbf {X}$ using sliding time window ($W = 5$), (**b**) GENIE3 feature ranking technique employed to learn influence of $\mathbf {X}_{-1}$ on node 1, (**c**) Directed acyclic graph showing relative order of 5 zones based on topological sorting of dates, and (**d**) Inflow and outflow based on weighted degree centrality of node 4.

### Network inference

Let us discuss the features of the network inference approach. Given a time window of *W* days, let $\mathbf {X} = \{x_1, x_2, \ldots , x_n \}$ (shown in Fig. 1a), where *n* is the number of zones and $x_u = [x_u^{t_{low}}, x_u^{t_{low} + 1}, \ldots , x_u^{t_{low} + W - 1}]$ is the data corresponding to the $u-$th zone (i.e., $x_u^t$ is the measurement of zone *u* on day $t \in [t_{low}, t_{high}]$) (where $t_{high} = t_{low} + W - 1$). Given any pair of zones *u* and *v* (s.t. $0 \le u, v \le n$), the purpose of network inference is to predict from $\mathbf {X}$ how *u* influences *v* ($u \not = v$), and vice versa. We employ GENIE3^28^ (which was conceived to derive the regulatory information among genes from expression data) to learn the influence of each zone (instead of gene) on another in terms of contagion spread. GENIE3 functions on the assumption that any $x_u^t$ is a function *f* of the expression of $x_v$ ($u \not = v$) plus random noise $\epsilon$, i.e.,

1 $$\begin{aligned} x^{t}_u = f(\mathbf {X}^{t}_{-u}) + \epsilon _t \forall t \in [t_{low}, t_{high} - 1]  \end{aligned}$$

Here $\mathbf {X}^{t}_{-u}$ is the vector containing the *t*-th measurement of all vectors except $x_u$, i.e.,

2 $$\begin{aligned} \{x_1, x_2, \ldots , x_{u-1}, x_{u+1}, \ldots , x_n \} \end{aligned}$$

At each time interval starting at $t_{low}$, GENIE3 solves Eq. (1) to calculate $\mathbf {W} \in \mathbb {R}^{n \times n}$, where each element $w_{u,v} \in [0, 1]$ captures the influence of node *u* on node *v*^28^. Specifically, for each node *v*, GENIE3 employs machine learning feature ranking technique to find confidence value $w_{u, v}$ that minimizes the squared error $\sum _t (x^{t}_v - f(\mathbf {X}^{t}_{-v}))^2$ (shown in Fig. 1b). Matrix $\mathbf {W}$ can be represented as a fully-connected, network *G* of *V* nodes, where $w_{u, v}$ is the weight of the directed link (*u*, *v*).

#### Temporal influence

Given any window size $W \in \mathbb {Z}$, we generate a series of time-varying influence networks, capturing the evolution of the influence of each zone on another. Given $t_{high} = t_{low} + W - 1$, each temporal network $G_{t_{low} \rightarrow t_{high}}$ is a fully-connected graph constructed on daily infection numbers of all zones over a period $[t_{low}, t_{high}]$ by GENIE network inference algorithm. It comprises of |*V*| zones as nodes and directed weighted links $w_{u,v}$ for each pair of $u, v \in V$ ($u \not = v$). We apply a sliding window to calculate a series of temporal influence networks $G_{1 \rightarrow W}, G_{2 \rightarrow W + 1}, G_{3 \rightarrow W + 2}, \ldots , G_{T\rightarrow T + W - 1}$ to understand the evolving inter-zone influence. Furthermore, we adapt the notion of topological sorting^29^ to generate a graph of the relative ordering of the zones $V = \{1, 2, \ldots , n\}$ based on the dates associated with each zone *u*, *D*(*u*). We employ Algorithm 1 that finds a directed acyclic graph of groups of zones ranked in the increasing order of their first documented infection dates ($z_s$). The DAG $\Gamma$ has links between successive batches of zone nodes. Figure 1c shows the DAG from the dates of 5 zones.

#### Spatial influence

For each instance of influence network, we gauge the influence of each zone on its neighbor resulting in flow (i.e., inflow and outflow) of infection. Inflow and outflow for a node indicates the amount of traffic entering and leaving that node in a given directed network. Nalluri et al.^30^ proposed an influence diffusion model in a miRNA-miRNA regulation network, where each node (miRNA) weight is the expression score and the weights of the links leaving a node determine its influence on the neighboring miRNA. In our context, we calculate the traffic inflow and outflow of all zones based on the influence weights of the temporal network and daily infection counts. Specifically, we posit that each zone *u* has a weight $\phi (u)$, which is equal to the total daily infection count within time window $W = [t_{low}, t_{high}]$.

1. *Weighted in-degree centrality.* It is the traffic flowing into a node and is calculated as $F^{-}(u) = \sum _{v \in V} w_{v,u} \times \phi (v)$, where $w_{u, v}$ is the weight on the directed link (*u*, *v*) in the temporal network.
2. *Weighted out-degree centrality.* It is the traffic flowing out of a node and is calculated as $F^{+}(u) = \phi (u) \times \sum _{v \in V} w_{u,v}$, where $w_{u, v}$ is the weight on the directed link (*u*, *v*) in the temporal network.

We argue that the zones with high $F^{+}(u) - F^{-}(u)$ is one with a high inflow but a low outflow of contagion and is likely to have a surge in infection in future time intervals, while nodes with high weighted out-degree centrality ($F^{+}(u)$) are good spreaders. In Fig. 1d, we illustrate the weighted in- and out-degree centrality for node labelled 4 in a 7-node graph. Following this, we also employ the following measures to estimate the similarity in contagion profiles of zones. Consider any two weighted directed graphs $G_i (V, E_i)$ and $G_j (V, E_j)$.

**In-degree neighborhood similarity** Given any node $u \in V$, let vectors $a^u = \{w_{v,u} :(v,u) \in E_i\}$ and $b^u = \{w_{v,u} :(v,u) \in E_j\}$. Then, in-degree neighborhood similarity $S_{i,j}^{-}(u) = cos(a^u, b^u)$.

**Out-degree neighborhood similarity** Given any node $u \in V$, let vectors $a^u = \{w_{u,v} :(u,v) \in E_i\}$ and $b^u = \{w_{u,v} :(u,v) \in E_j\}$. Then, out-degree neighborhood similarity $S_{i,j}^{+}(u) = cos(a^u, b^u)$.
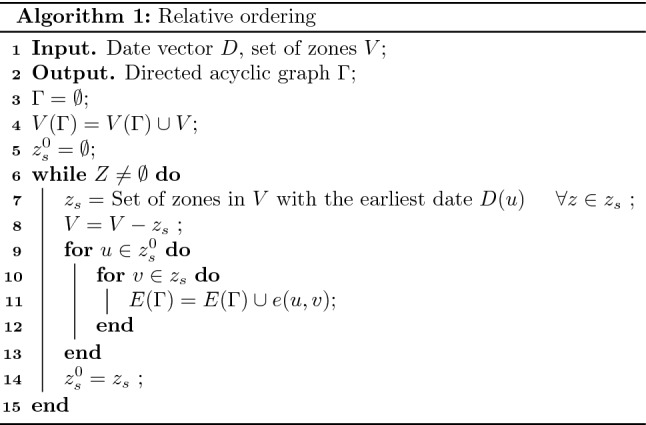


### Metrics

We employ the following similarity metrics.

#### Cosine similarity

It is the similarity between two vectors *a* and *b* on a scale of 0 and 1, calculated as the cosine angle between them, i.e., $cos(a, b) = \frac{a.b}{||a||.||b||}$. We calculate cosine similarities between two temporal networks $G_i$ and $G_j$ (where *i*, *j* are both time intervals) as $cos(\mathbf {v}_i, \mathbf {v}_j)$, where $v_i = \{w_{u,v}; \forall u,v \in V(G_i) \}$ and $v_j = \{w_{u,v}; \forall u,v \in V(G_j) \}$. Cosine similarities between temporal networks $G_{t \rightarrow t + W - 1}$ and $G_{t + 1\rightarrow t + W}$ capture the overall variation in mutual interaction among zones across time intervals $[t, t + W - 1]$ and $[t + 1, t + W]$.

#### Pearson correlation coefficient

It measures the strength of a linear association between two vectors, where correlation 1 is a positive correlation and $-1$ is perfect negative correlation.

## Results

We consider two scenarios, namely the counties of New York (NY) and US states. We utilize the counts and dates of COVID-19 daily infection in the (1) NY counties from 1st March to November 4 (data shared on https://github.com/satunr/COVID-19/blob/master/US-COVID-Dataset/county_daily_inf_(spatio-temp).csv) and (2) states from 21st January to 4 November, 2020 (https://github.com/satunr/COVID-19/blob/master/US-COVID-Dataset/us-states_cumulative_(spatio-temp).csv). The temporal influence networks for the NY state counties and US states (as discussed in “Network inference”) are shared as animations (in .gif format) in https://github.com/satunr/COVID-19/tree/master/NetworkInference, along with the Python script that were used to generate them. The experimental results are organized into the following four subsections: (1) phases in the pandemic timeline and (2) interaction among neighboring zones.

**Figure 2:**
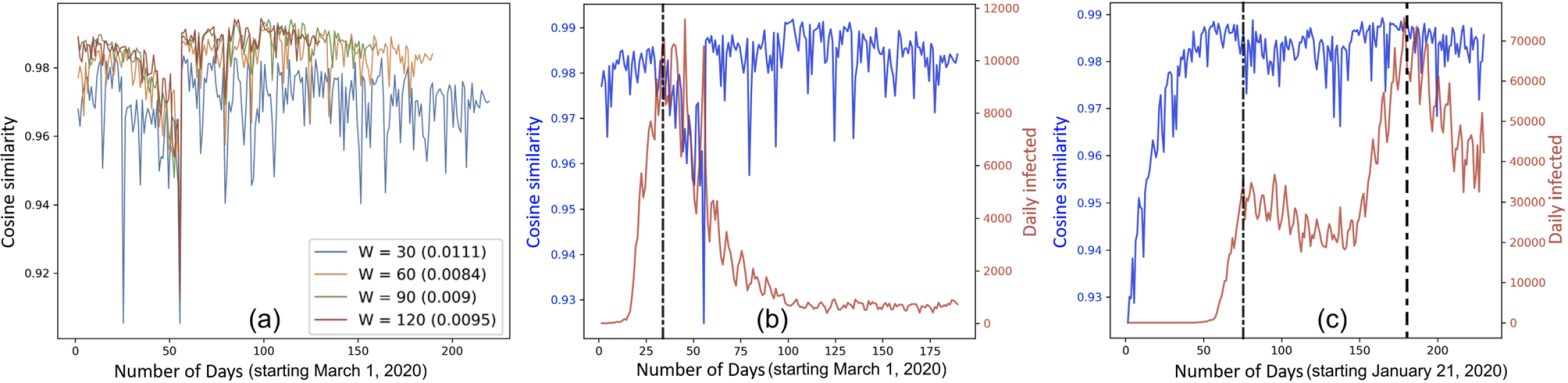
Phases in the pandemic timeline. (**a**) Cosine similarities of consecutive temporal influence networks generated from the NY county COVID-19 infection data for varying time windows (*W*); Cosine similarities between consecutive temporal networks and total daily infected numbers for $W = 60$ for (**b**) NY state counties (starting 1st March, 2020) and (**c**) US states (starting 21st January, 2020), with the black dotted lines indicating phase shifts.

### Phases in the pandemic timeline

In Fig. 2a, we generate temporal networks using GENIE3 (as described in “Network inference”) on the NY counties. We consider window sizes $W = 30, 60, 90, 120$ days and plot the cosine similarity between consecutive pairs of temporal networks (using approach described in “Metrics”), while noting the standard deviations in the corresponding curves equal to 0.0111, 0.0084, 0.0090 and 0.0095. Note that the cosine similarity curves for all window sizes follow a similar trend. Subsequently $W = 60$, which exhibits the least standard deviation, is used in subsequent experiments. We discuss the implications of variations in window size later in “Discussions”.

**Figure 3:**
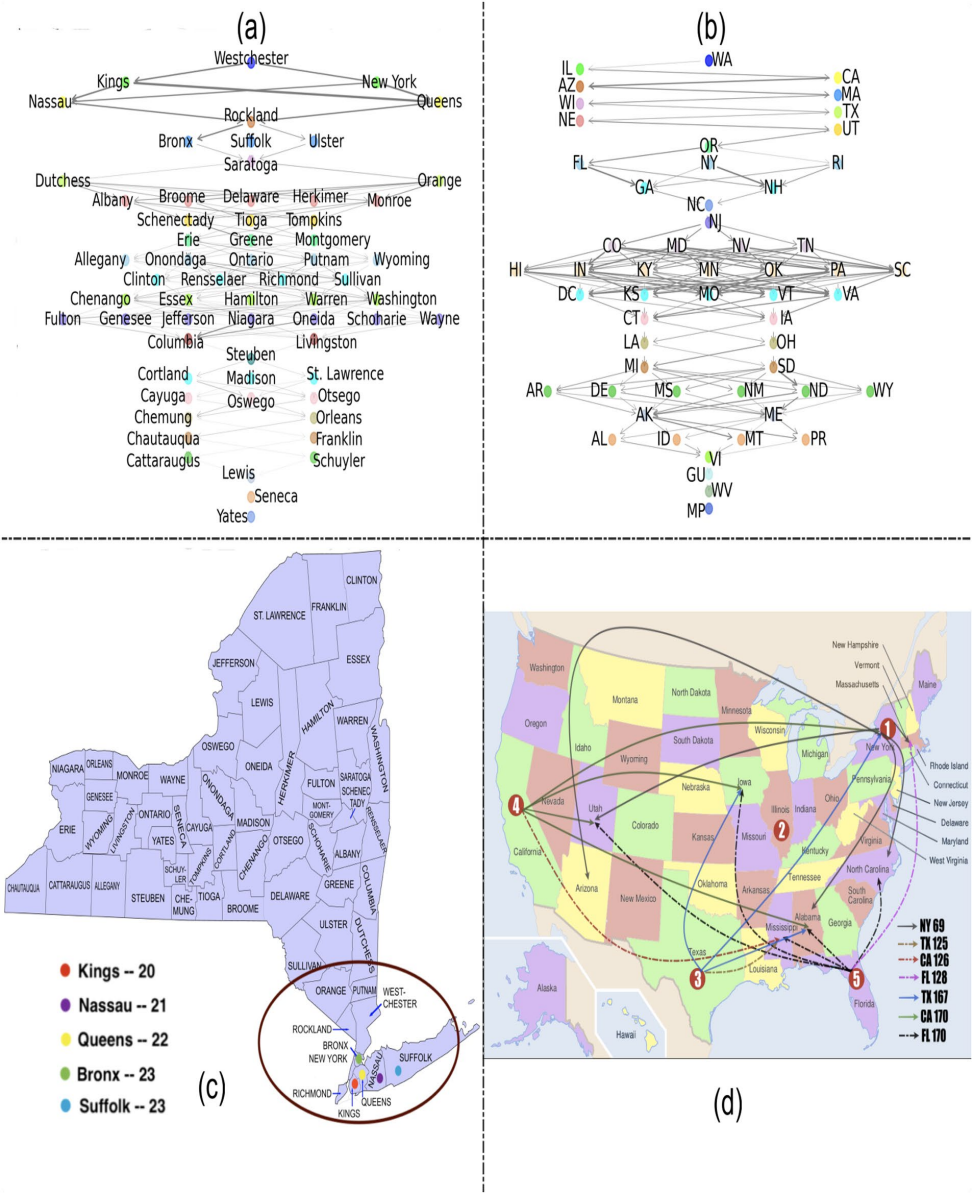
Interaction among neighboring zones. (**a**,**b**) Directed acyclic graph (DAG) of NY counties (Queens, Kings, Bronx, Nassau and Suffolk) and US states (New York, Illinois, Texas, California and Florida). The position of a zone (and not the links per se) on the DAG is important as they represent the relative order based on the first date of infection; (**c**,**d**) USA map showing the counties and states with strong interaction, with the circles marking regions with high spread. The legends represent the day (starting on March 1st and January 21, 2020 starting in NY counties and US states, respectively) when the daily infection peaked. Each black directed dotted line ($u \rightarrow v$) in (**d**) shows the high Pearson correlation coefficient ($\ge 0.7$) between the weighted out-degree centrality ($F^{-}(u)$) of spreader zone *u* curve and any weighted in-degree centrality—out-degree centrality ($F^{+}(u) - F^{-}(u)$) curve of affected zone *v* over a 10-day period starting when the $F^{-}(u)$ curve of *u* reached its peak. The numbers in the red circles in (**d**) represent the level of the corresponding states (and their neighbor states) in the DAG.

Fig. 2b,c show the cosine similarities between consecutive temporal networks for NY counties and US states, along with the daily total infected numbers, for $W = 60$. We intuit that the drop in similarity is indicative of a phase shift in the interaction (determined by weights $w_{i,j}$) among zones. The sharp rise and fall in total daily infected count in the NY counties (in Fig. 2b) correspond to the brief decline in cosine similarity roughly at day 30 (shown in black dotted line), suggesting a change in mutual interaction among zones. Conversely, despite the rise and fall in daily infected numbers, the cosine similarities of the temporal networks derived from the US states undergo a much smaller dip—showing phase shifts in COVID timeline—at around day 70 and later at day 180 (shown as dotted lines in Fig. 2b,c).

### Interaction among neighboring zones

We next trace the path of contagion through the zones (i.e., NY counties and US states) during the different stages of the COVID-19 timeline.

#### First wave

For the first wave of contagion across zones, we rank the zones in the increasing order of their first documented infection date and calculate the directed acyclic graphs (DAGs) of zones (refer to “Network inference”). The opaqueness of directed edge in the DAG from zone *u* to *v* is proportional to the mean edge weight of the temporal networks from *u* to *v*. For NY counties and US states, the edges with weights ($w_{u,v}$) $\ge 0.05, 0.07$, respectively, are preserved in the DAGs.

Figure 3a shows that the zone-wise interaction are the strongest from Westchester $\rightarrow$ Kings, New York $\rightarrow$ Nassau, Queens $\rightarrow$ Rockland. Figure 3c (depicting counties marked on a map attributed to Andre Koehne, https://commons.wikimedia.org/wiki/File:New_York_Counties.svg, via Wikimedia Commons) shows that Westchester, Kings, New York, Nassau, Queens and Rockland are neighboring counties serving as gateway and spreaders of contagion into New York state. Similarly, Fig. 3b depicts that Washington showed one of the first cases of COVID-19. However, the flow of contagion is the strongest from New Jersey $\rightarrow$ Colorado, Maryland, Nevada, Tennessee $\rightarrow$ Hawaii, Indiana, Kentucky, Minnesota, Oklahoma, Pennsylvania, South Carolina $\rightarrow$ Columbia, Kansas, Missouri, Vermont Virginia. Figure 3d (depicting US states marked on a map attributed to Eric Pierce, https://commons.wikimedia.org/wiki/File:Map_of_USA_showing_state_names.png, via Wikimedia Commons) shows that few neighboring states are one level apart or on the same level in the DAG, namely, (1) Montana, Wyoming, North Dakota and South Dakota, (2) Missouri and Iowa, (3) New Jersey and Nevada, (4) Kentucky, Tennessee, South Carolina, are neighbors on the US map, (4) California, Arizona and (5) Michigan, Ohio. The labels on both Fig. 3c,d have been added using Adobe Photoshop CS6 (https://www.adobe.com/products/photoshop.html).

#### Subsequent waves

**Figure 4:**
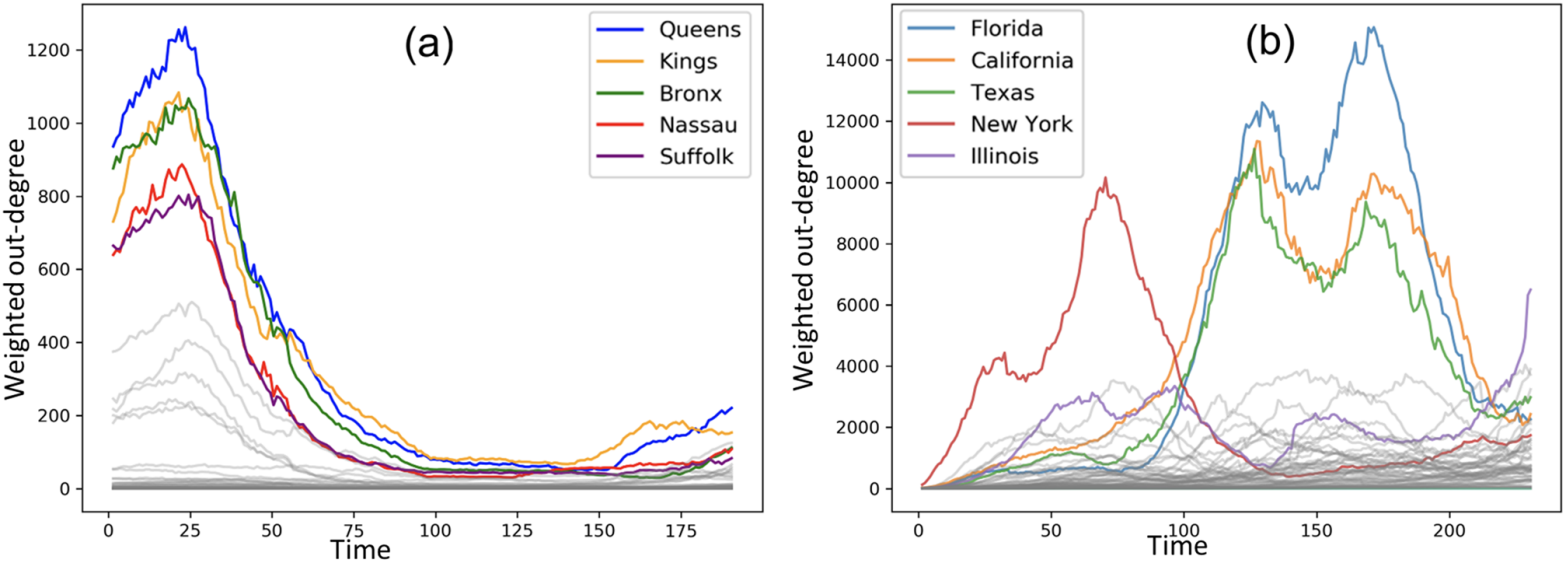
The 5 zones, i.e., (**a**) NY counties and (**b**) US states exhibiting the highest weighted out-degree centrality (and thereby acting as spreaders) in the temporal networks.

We identify the spreader zones (by calculating the weighted out-degree centrality as defined in “Network inference”) on the temporal networks derived from the NY counties and US state infection data. Figure 4a shows that Queens, Kings, Bronx, Nassau and Suffolk emerge as the counties with the highest out-degree centrality while New York, Illinois, Texas, California and Florida are states with the highest out-degree centrality. The spreader counties as well as states are highlighted in red circles in Fig. 3c,d. Moreover, it is worth noting that the out-degree centralities of counties peak at nearly the same time, whereas the out-degree centrality of states peak at different timepoints. This suggests that, unlike the spreader states, there is a strong mutual interaction among the spreader counties. To validate this, we record the set of zones that each spreader zone influences the most when its out-degree centrality peaks. Specifically, we pick out temporal network at each timepoint when a spreader zone *u* has the highest weighted out-degree centrality. Following this, we identify the zone(s) *v* that are highly influenced by *u*, i.e., $w_{u,v}$ is the highest. We report such zones and corresponding $w_{u,v}$ in Table 1.

In Table 1 we summarize adequate evidence of the notion that there is a strong mutual influence among the spreader counties (Queens, Kings, Bronx, Nassau, Suffolk). Also, the weighted out-degree centrality of the spreaders subsided after day 75, while showing another sign of rising after day 150, marking the start of another wave. On the other hand, there is little mutual interaction among the US states. The strongest interaction, with the exception of Florida $\rightarrow$ South Carolina, Texas $\rightarrow$ Oklahoma, New York $\rightarrow$ New Jersey, Illinois $\rightarrow$ Indiana, exist between the spreader states and relatively distant states. This suggests that at a state level, the spread of infection takes place via longer trips during the subsequent stages of the COVID-19 pandemic.

#### Zone-wise variation of neighborhood in temporal networks

Recall that the series of temporal influence networks are obtained by applying GENIE3 on the daily infection count of zones at specific time intervals in the COVID-19 timeline (see “Network inference” for details). Each temporal network is directed, fully-connected and weighted with each node (representing a zone) having a set of incoming and outgoing edge weights from all other nodes defined as in- and out-neighborhood respectively in “Graph theory preliminaries”. The change in in- and out-neighborhood of a zone indicates the variation in the extent to which the zone is influenced by or influencing contagion in other nodes.

**Table 1:** The set of zones (counties and states) that each spreader zone influences the most in the temporal network where its out-degree centrality peaks (single or multiple times).

Spreader zone	Influenced zone and weight
*NY counties*	
Queens	(Kings, 0.10), (Bronx, 0.10), (Nassau, 0.09)
Kings	(NY, 0.11), (Bronx, 0.11), (Queens, 0.11)
Bronx	(Kings, 0.08), (NY, 0.08), (Suffolk, 0.08)
Nassau	(Suffolk, 0.11), (Queens, 0.10), (Richmond, 0.10)
Suffolk	(Richmond, 0.12), (Nassau, 0.11), (Bronx, 0.08)
*US states*	
FL peak 1	(AZ, 0.12), (SC, 0.10), (NM, 0.10)
FL peak 2	(SC, 0.11), (OH, 0.09), (NV, 0.09)
CA peak 1	(GA, 0.11), (TN, 0.09), (MS, 0.09)
CA peak 2	(SD, 0.11), (ID, 0.09), (ND, 0.09)
TX peak 1	(OK, 0.07), (AZ, 0.07), (MS, 0.06)
TX peak 2	(ID, 0.11), (CA, 0.06), (SD, 0.06)
NY	(NJ, 0.13), (MN, 0.13), (MI, 0.08)
IL peak 1	(CO, 0.08), (NJ, 0.08), (OH, 0.07)
IL peak 2	(CN, 0.07), (IN, 0.07), (MD, 0.06)

For a node *u*, we find weight vectors of in- and out-neighborhoods for graph $G_{t, t + W - 1}$ as $V_{t, t + W - 1}^{\mathrm{{in}}}(u) = \{ w_{v, u}; \forall v \in V\}$ and $V_{t, t + W - 1}^{\mathrm{{out}}}(u) = \{ w_{u, v}; \forall v \in V\}$. We determine the neighborhood variation of *u* by calculating the mean cosine similarity (see “Metrics”) between its in- (or out-neighborhood) vector between pairs of consecutive temporal networks $G_{t, t + W - 1}$ and $G_{t + 1, t + W}$, i.e.,

For in-neighbors:

3 $$S_{\mathrm{{in}}} (u) = \frac{1}{T - W + 1} \sum _{t = 1}^{T - W + 1} \cos (V_{t , t + W - 1}^{\mathrm{{in}}} (u), V_{t, t + W}^{\mathrm{{in}}} (u)) $$

For out-neighbors:

4 $$\begin{aligned} S_{\mathrm{{out}}} (u) = \frac{1}{T - W + 1} \sum _{t = 1}^{T - W} \cos (V_{t + 1, t + W}^{\mathrm{{out}}} (u), V_{t + 1, t + W}^{\mathrm{{out}}}(u)) \end{aligned}$$

**Figure 5:**
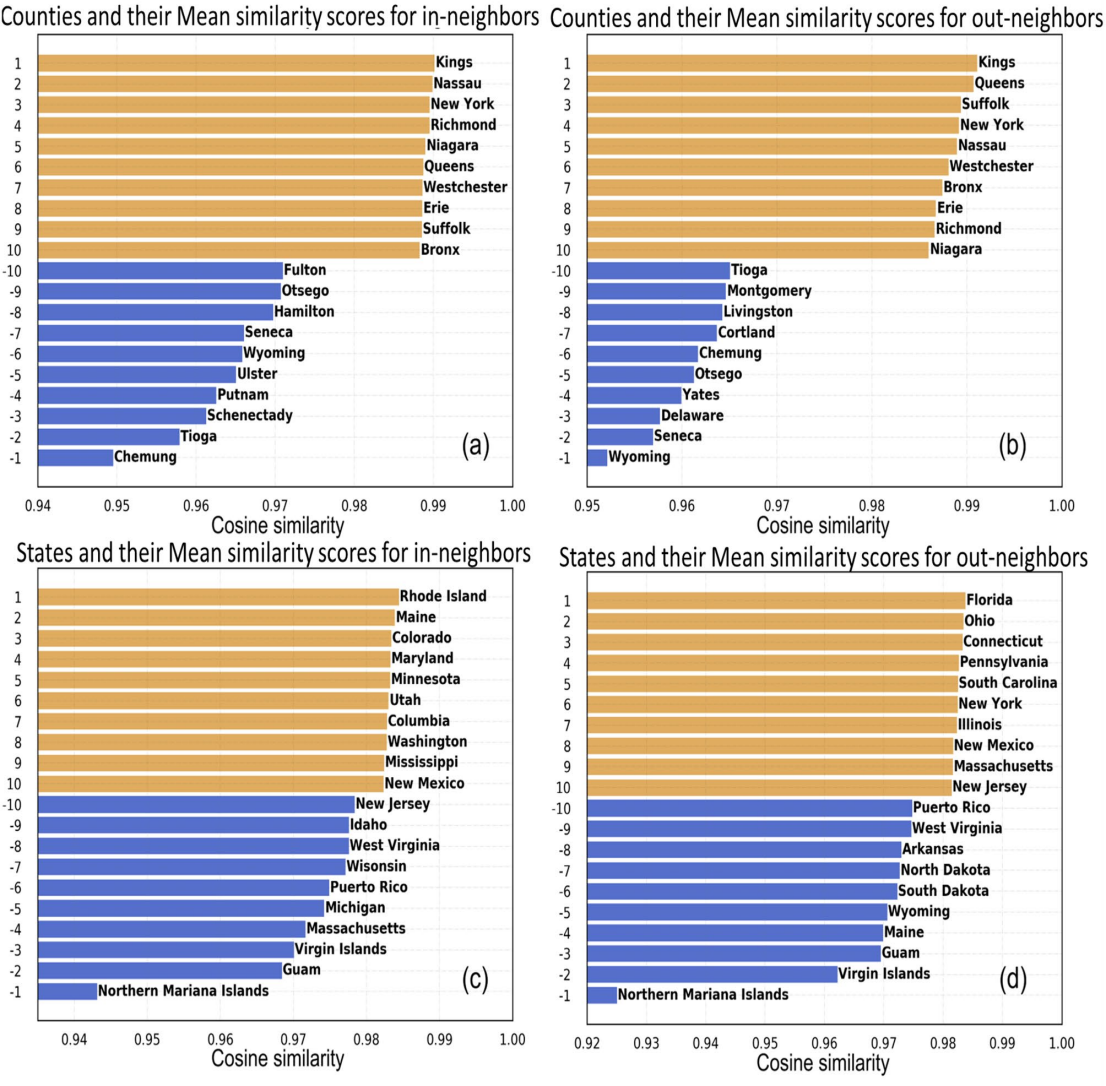
Zone-wise temporal variations in the neighborhood of influence. Cosine similarity on consecutive temporal networks to determine the counties with highly similar (shown in orange) and dissimilar (shown in blue) (**a**) in-neighborhood (**b**) out-neighborhood; US states with highly similar and dissimilar (**c**) in-neighborhood (**d**) out-neighborhood.

Figure 5a shows that the key spreader counties Kings, Nassau, Queens, Suffolk and Bronx exhibit the highest similarity in the in- and out-neighborhoods, reaffirming the strong mutual interaction between them as we reported in Table. 1. Lesser infected counties such as Tioga, Chemung, Otsega, Seneca and Wyoming have a highly variable neighborhood. For the US states, the same zones do not have the highest (or least) in- and out-neighborhood. While the spreader states do not feature among zones with similar in-neighbors, three spreader states (Florida, New York and Illinois) have similar outgoing neighbors, implying that they influence similar set of states (Fig. 5b).

#### Most affected zones

**Figure 6:**
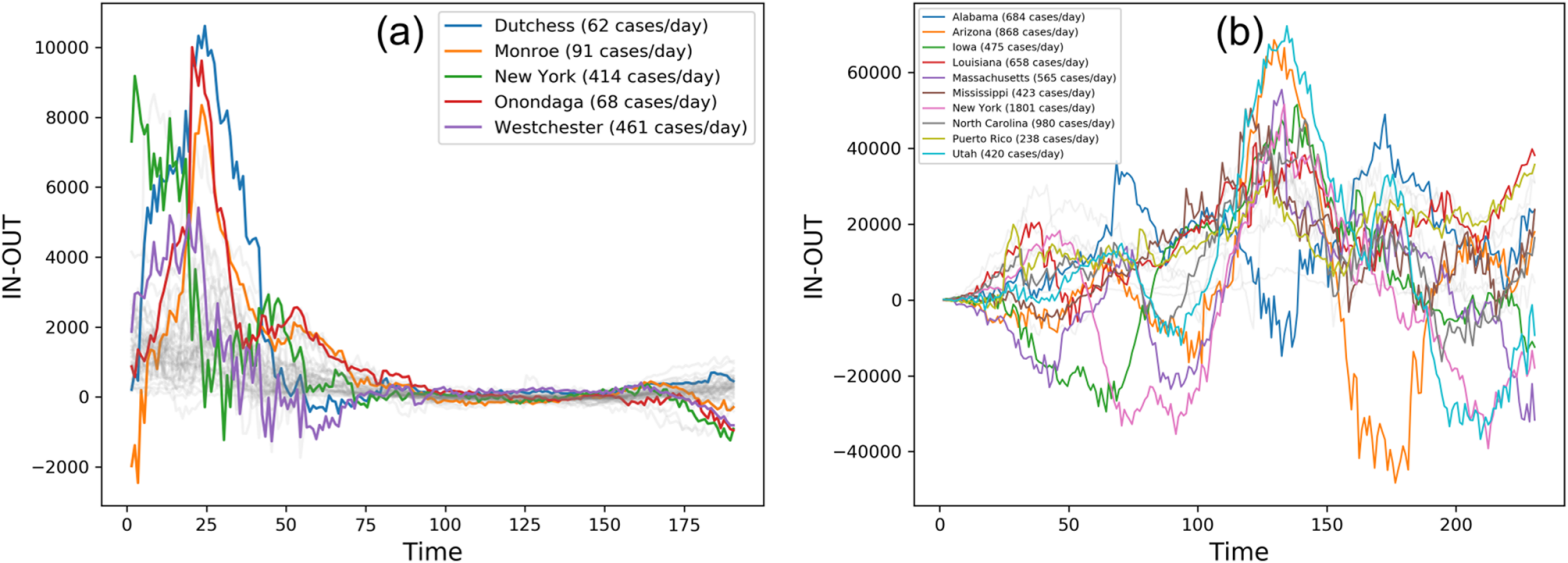
Most affected zones with the corresponding COVID-19 daily infection numbers. (**a**) NY counties and (**b**) US states showing a high difference between inflow and outflow measured in terms of difference between weighted out-degree from weighted in-degree.

We hint in “Network inference” that the zones with high inflow but low outflow, i.e., $F^{+}(u) - F^{-}(u)$ are likely to be the most affected by the pandemic. We show the counties and US states with the highest peaks in $F^{+}(u) - F^{-}(u)$. Figure 6a shows that Dutchess, Monroe, New York, Onondaga and Westchester are the most affected with 62, 91, 414, 68 and 461 mean cases per day between day 0 and day 90. Earlier we have shown that most of the highly infected counties (viz., Dutchess, Monroe, New York and Westchester) are placed at the top of the DAGs in Fig. 3a showing the first affected counties. Just as different US states (namely, New York, Illinois, Florida, California and Texas) have been shown (in Fig. 4b) to act as spreaders at different timepoints, Fig. 5b shows high temporal heterogeneity in highly affected states. Alabama, Arizona, Iowa, Louisiana, Massachusetts, Mississippi, New York, North Carolina, Puerto Rico and Utah all undergo wavelike rise and fall in $F^{+}(u) - F^{-}(u)$ and register significantly high daily infection counts over the COVID-19 timeline and has been recorded in brackets in Fig. 6b.

We take this analysis a step further by tracing the path of contagion by analysing the relationship between the spreader and the most affected US states. For any zone *u*, let $C_{t_1 \rightarrow t_2}^{out}(u)$ and $C_{t_1 \rightarrow t_2}^{in - out}(u)$ be vectors of $F^{-}(u)$ and $F^{+}(u) - F^{-}(u)$ between time interval $[t_1, t_2]$. For each peak (say, at time $t_1$) in the $F^{+}(u)$ curve of a US state spreader *u* in Fig. 4b, we calculate the Pearson correlation coefficient (see “Metrics”) between $C_{t_1 \rightarrow t_1 + k}^{out}(u)$ and $C_{t_1 \rightarrow t_1 + k}^{in - out}(v)$, where *k* is the time period of observation and *v* is an affected state shown in Fig. 5b. We posit that the high correlation ($\ge 0.7$) between each $F^{-}(u)$ spreader curve and any $F^{+}(u) - F^{-}(u)$ curve of affected zones over the $k =$ 10-, 20- and 30-day period, may be a consequence of the influence of the spreader on that affected zone and help trace the path of contagion.

Table 2 shows the (mean, standard deviation) in Pearson correlation coefficient for the spreader vs. affected zone curves over $k = 10, 20, 30$; and correlation $\ge 0.7$ is marked in red. (Individual correlation tables for $k = 10, 20, 30$ are provided as supplementary materials; the correlations do not vary significantly across tables.) While Florida (FL) and New York (NY) emerge as the strongest spreaders, since the table only represents the major spreader and affected states, there are some spreaders (shown as columns with peak day in the COVID-19 timeline) and affected zones (rows) that do not participate in high correlation. It is worth noting that the correlation between NY spreader and NY affected zone is negative ($-0.58$). Albeit counter-intuitive, this is because we are capturing the relationship between the potential of a zone to act as spreader (i.e., outflow) and another zone receiving the contagion (i.e., inflow - outflow) and it is not possible for NY to act as the spreader and also as the affected zone at the same time. We represent the high correlations with directed black dotted lines from spreaders to affected zones Fig. 3d. There are only a few directed links between neighbor states, suggesting that long distance (and not short distance) trips are major means of contagion spread among the US states.

## Discussions

The experimental results provide several insights into the dynamics of COVID-19 spread. First, the cosine similarity analysis of consecutive temporal network snapshots show that the pairwise influence among zones change with the variations in overall infection count. Although the drop in cosine similarity is more pronounced in case of a smaller region (namely, NY counties) than for a larger region (namely, US states), the dissimilarity, which may vary in extent depending on scale or region, in the temporal networks can be a measure of phase shifts in the pandemic timeline. Second, in a smaller region like NY state, there are neighboring counties (like Queens, Kings, Bronx, Suffolk and Nassau) that exhibit high mutual influence (see Table 1) that remain largely invariant over time (Fig. 5c,d). This suggests that travel restrictions between counties or setting up quarantine zones by clustering the neighboring high mutual influence zones can provide effective policy making avenues to curb pandemic spread. On the other hand, upon consideration of the larger scenario comprising all the states of USA, only a few neighboring states are influencing the contagion spread into one another. This suggests that the overall spread of infection is a consequence of essential long-distance trips in bulk as opposed to short distance trips between neighbor states; such long distance trips are more likely to be an outcome of air-traffic between different states in the USA. Furthermore, Fig. 2c shows that the change in infection counts are not always reflected by the dissimilarity of temporal networks depicting US states at different timepoints; this is in contrast to the effects seen in Fig. 2b where the phase shift is significantly more pronounced. This suggests that travel restrictions imposed between US states based on their instantaneous infection counts may not turn out to be an effective public policy strategy of mitigating contagion spread; instead the high contagion spread edges from the directed graphs inferred by our proposed method may serve as a better indicator of devising such restrictions in the future.

**Table 2:** (Mean, standard deviation) of Pearson correlation between each weighted out-degree centrality ($F^{-}(u)$) spreader (column) curve and a weighted in-degree centrality - out-degree centrality ($F^{+}(u) - F^{-}(u)$) curve of affected zones (row) over 10-, 20- and 30-day windows, where correlation $\ge 0.7$ is marked in red.

**State**	FL(170)	FL(128)	CA(126)	CA(170)	TX(125)	TX(167)	NY(69)	IL(95)
AL	**0.88**, 0.07	-0.34, 0.43	-0.51, 0.54	**0.82**, 0.09	-0.34, 0.42	**0.72**, 0.25	**0.87**, 0.13	0.29, 0.62
AZ	-0.49, 0.73	0.61, 0.41	0.65, 0.40	-0.49, 0.70	0.51, 0.38	-0.38, 0.7	**0.9**, 0.02	-0.83, 0.13
IA	**0.84**, 0.08	-0.15, 0.43	0.34, 0.28	**0.89**, 0.04	0.11, 0.38	**0.82**, 0.06	-0.96, 0.02	-0.55, 0.24
LO	-0.50, 0.17	-0.43, 0.27	0.04, 0.4	-0.66, 0.06	-0.16, 0.46	-0.63, 0.12	-0.74, 0.13	-0.74, 0.16
MA	0.48, 0.31	**0.77**, 0.12	0.66, 0.23	0.52, 0.28	0.44, 0.42	0.41, 0.3	**0.89**, 0.09	-0.89, 0.13
MI	**0.72**, 0.25	0.54, 0.41	**0.79**, 0.05	0.59, 0.24	**0.86**, 0.04	0.54, 0.32	-0.89, 0.07	-0.29, 0.67
NY	**0.82**, 0.17	0.54, 0.35	0.46, 0.35	**0.71**, 0.16	0.25, 0.53	**0.86**, 0.03	-0.58, 0.29	-0.88, 0.15
NC	**0.73**, 0.28	-0.18, 0.22	0.23, 0.35	0.64, 0.43	0.31, 0.41	0.62, 0.35	**0.72**, 0.32	-0.90, 0.08
PR	0.16, 0.54	**0.73**, 0.23	**0.92**, 0.06	0.07, 0.62	**0.92**, 0.03	0.30, 0.29	-0.25, 0.46	-0.85, 0.04
UT	**0.89**, 0.15	0.45, 0.28	0.44, 0.72	**0.84**, 0.19	0.18, 0.75	0.55, 0.63	**0.92**, 0.09	-0.91, 0.06

Third, the proposed approach considers the most affected zones are the ones with high inflow but low outflow (termed *in - out*) of contagion traffic measured in terms of weighted degree centrality. Interestingly, for NY counties as well as US states, a small subset of nodes emerge as highly affected. Figure 6 shows that Alabama, Arizona, Iowa, Louisiana, Mississippi, New York, North Carolina, Puerto Rico and Utah^31–35^ not only have high daily infection rates but also show a near-periodic rise and fall in in-out score over time. This goes to show that the same set of zones in a region may repeatedly emerge as most affected at varying stages in the pandemic period as a result of the combined influence of other zones. Fourth, it is worth asking how the proposed approach can be useful in the event of an ongoing pandemic when one does not have the time-series infection numbers for the entire cycle of the pandemic. Since the cosine similarity between consecutive temporal networks does not vary considerably over time (except when the daily infection count changes drastically), we posit that the current infection counts may be utilized to infer zone-level influence weights to inform travel restriction measures to contain mobility between the affected zones and other zones they influence heavily at a given time instance.

**Figure 7:**
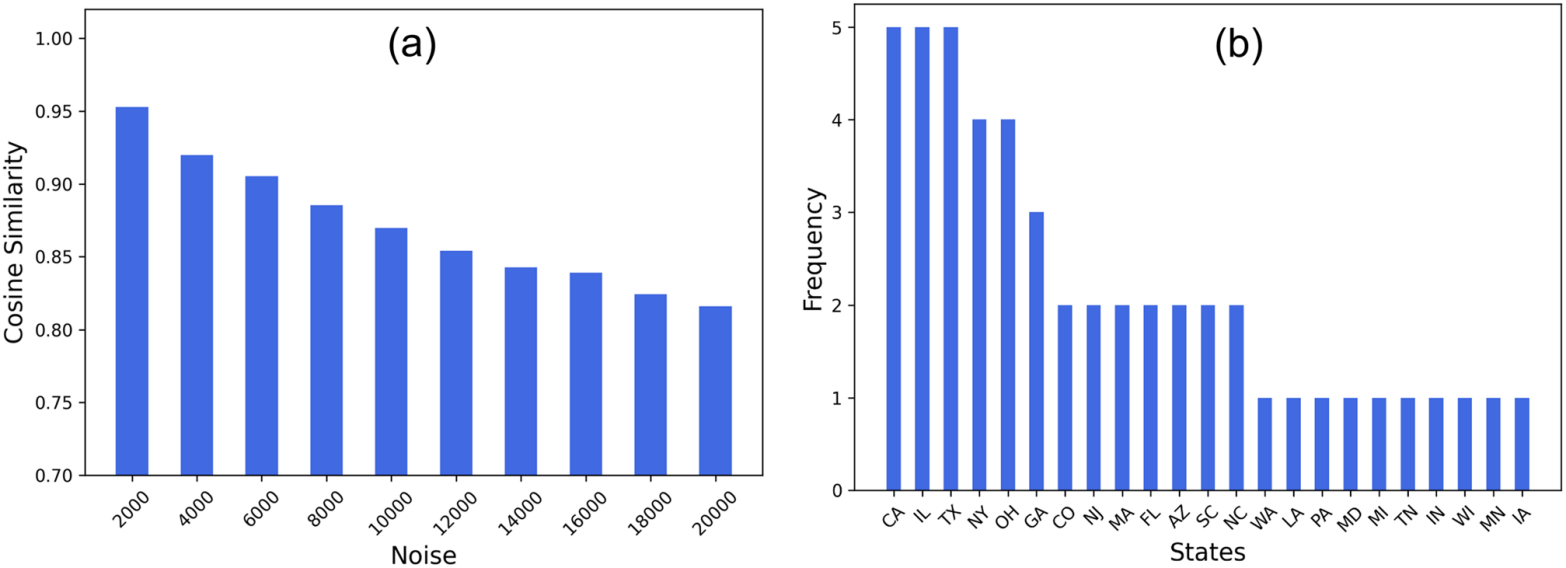
Effectiveness of network inference in predicting contagion. (**a**) Cosine similarity between original network on US infected data and noise data created by adding random noise; (**b**) Frequency of states emerging as top 10 spreaders in five influence networks (one pre-lockdown January to mid-March 2020 and four post-lockdown mid-March to November 2020).

Fourth, as we discuss in “Introduction” that the daily infected count is one of the accessible epidemiological information. To study the effect of minor discrepancy between the actual and reported infection count on the accuracy of the resultant influence network, we add random noise (sampled from normal distribution with mean 2000 to 20000 and standard deviation equal to $5\%$ of the mean noise) to the daily infected numbers of US states. Figure 7a shows that the cosine similarity between the network derived from the original infected numbers and its noisy counterpart is over 0.8, suggesting that the approach is fairly accurate despite approximations. Moreover, we generate the 5 influence networks i.e., one pre-lockdown (January to mid-March 2020) and four post-lockdown (each created with time window $W = 60$ days, namely, March 15–May 14, May 15th–July 14, July 15–September 14, September 15–November 10, 2020). Then, we calculate the top 10 spreader zones (with high out-degree centrality as discussed in “Network inference”). Figure 7b shows that specific US states like California, Illinois, Texas, etc. consistently emerge as top spreaders across the five influence networks. A network inference approach that leverages this information can thus be a highly robust way of understanding overall contagion patterns.

Finally, the choice of the window size (*W*) controlling the duration of infection data to be considered while generating the influence graph for a single timepoint may influence the findings. In Fig. 2a we showed that the overall trends in the cosine similarity is retained for varying $W = 30, 60, 90, 120$ days. While we used the $W = 60$ since it results in the least noisy similarity curve (calculated in terms of standard deviation), we shall explore a more comprehensive criterion to determine the ideal *W*. Given that too large a *W* will prevent the model from recognizing minor phase shifts in contagion and too small *W* may render the resultant influence graph uninformative, a more dynamic, adaptive approach, keeping socioeconomic and demographic factors in mind, may be useful in determining its value.

## Supplementary Information

Supplementary Information.
