# Supplementary material for: Spatiotemporal tracing of pandemic spread from infection data
Source: Sci Rep. 2021 Sep 3;11:17689. doi: 10.1038/s41598-021-97207-5 (PMC8417268; doi:10.1038/s41598-021-97207-5)
Supplement: Supplementary file 1 — Supplementary Information. [file 41598_2021_97207_MOESM1_ESM.pdf]

Spatio-temporal tracing of pandemic spread from infection data

Supplementary Materials

Satyaki Roy[a,], Preetom Biswas[b], Preetam Ghosh[c]

[a] *University of North Carolina, Chapel Hill, USA*
[b] *Arizona State University, Tempe, AZ, USA*
[c] *Virginia Commonwealth University, Richmond, USA*

1. Relationship between spreader and most affected zones

We trace the path of contagion by analysing the relationship between the spreader and most affected US states. For any zone u, let $C^{out}_{t_1 \to t_2}(u)$ and $C^{in-out}_{t_1 \to t_2}(u)$ be vectors of $F^-(u)$ and $F^+(u) - F^-(u)$ between time interval $[t_1, t_2]$. For each peak (say, at time t_1) in the $F^+(u)$ curve of a US state spreader, we calculate the Pearson correlation coefficient $C^{out}_{t_1 \to t_1+k}(u)$ and $C^{in-out}_{t_1 \to t_1+k}(v)$, where k is the time period of observation and v is an affected state. The high correlation (≥ 0.7) between each $F^-(u)$ spreader curve and any $F^+(u) - F^-(u)$ curve of affected zones over the $k =$ 10-, 20- and 30-day period may be a result of the influence of the spreader on that affected zone.

**State**	FL(170)	FL(128)	CA(126)	CA(170)	TX(125)	TX(167)	NY(69)	IL(95)
AL	0.88	0.12	0.10	0.77	0.13	0.44	0.94	-0.39
AZ	0.35	0.87	0.18	0.31	0.07	0.42	0.91	-0.68
IA	0.76	-0.60	0.09	0.90	-0.17	0.77	-0.92	-0.32
LO	-0.32	-0.74	-0.19	-0.68	-0.29	-0.61	-0.72	-0.57
MA	0.19	0.81	0.40	0.27	-0.03	0.11	0.79	-0.74
MI	0.82	0.72	0.73	0.52	0.81	0.20	-0.80	0.46
NY	0.94	0.73	0.06	0.72	-0.34	0.85	-0.87	-0.70
NC	0.40	-0.43	0.23	0.14	0.53	0.22	0.86	-0.81
PR	-0.45	0.84	0.84	-0.64	0.89	0.05	-0.74	-0.83
UT	0.71	0.46	-0.39	0.62	-0.67	-0.17	0.97	-0.84

Table 1: Pearson correlation between each weighted out-degree centrality ($F^-(u)$) spreader (column) curve and a weighted in-degree centrality - out-degree centrality ($F^+(u) - F^-(u)$) curve of affected zones (row) over 10-day periods, where correlation ≥ 0.7 is marked red

Tables 1, 2 and 3 show the Pearson correlation coefficient between each weighted out-degree centrality ($F^-(u)$) spreader (column) curve and a weighted

in-degree centrality - out-degree centrality ($F^+(u) - F^-(u)$) curve of affected zones (row) over 10-, 20-, 30-day periods. In general, although there is a considerable overlap in positive correlations (marked red) across the corresponding entries, the number of high positive coefficients (≥ 0.7) increases with period size.

**State**	FL(170)	FL(128)	CA(126)	CA(170)	TX(125)	TX(167)	NY(69)	IL(95)
AL	0.81	-0.73	-0.79	0.76	-0.49	0.80	0.95	0.48
AZ	-0.87	0.81	0.91	-0.86	0.67	-0.67	0.88	-0.87
IA	0.83	0.24	0.27	0.85	-0.035	0.80	-0.98	-0.52
LO	-0.50	-0.29	-0.18	-0.59	-0.52	-0.52	-0.62	-0.89
MA	0.44	0.86	0.75	0.45	0.60	0.38	0.98	-0.95
MI	0.89	0.83	0.83	0.85	0.89	0.83	-0.94	-0.95
NY	0.63	0.75	0.57	0.54	0.43	0.82	-0.57	-0.95
NC	0.85	-0.05	-0.13	0.84	-0.16	0.77	0.94	-0.94
PR	0.43	0.88	0.94	0.36	0.93	0.23	0.15	-0.82
UT	0.97	0.73	0.86	0.92	0.49	0.88	0.98	-0.94

Table 2: Pearson correlation between each weighted out-degree centrality ($F^-(u)$) spreader (column) curve and a weighted in-degree centrality - out-degree centrality ($F^+(u) - F^-(u)$) curve of affected zones (row) over 20-day periods, where correlation ≥ 0.7 is marked red

**State**	FL(170)	FL(128)	CA(126)	CA(170)	TX(125)	TX(167)	NY(69)	IL(95)
AL	0.94	-0.42	-0.85	0.92	-0.65	0.91	0.72	0.79
AZ	-0.95	0.14	0.86	-0.94	0.77	-0.88	0.92	-0.91
IA	0.92	-0.06	0.64	0.91	0.55	0.89	-0.96	-0.79
LO	-0.67	-0.26	0.50	-0.70	0.35	-0.76	-0.88	-0.76
MA	0.81	0.63	0.84	0.82	0.75	0.72	0.90	-0.98
MI	0.44	0.06	0.80	0.38	0.88	0.60	-0.92	-0.80
NY	0.88	0.14	0.74	0.86	0.67	0.89	-0.28	-0.98
NC	0.93	-0.06	0.57	0.92	0.56	0.87	0.35	-0.95
PR	0.51	0.47	0.96	0.48	0.94	0.62	-0.15	-0.89
UT	0.99	0.17	0.85	0.97	0.71	0.95	0.82	-0.96

Table 3: Pearson correlation between each weighted out-degree centrality ($F^-(u)$) spreader (column) curve and a weighted in-degree centrality - out-degree centrality ($F^+(u) - F^-(u)$) curve of affected zones (row) over 30-day periods, where correlation ≥ 0.7 is marked red
